# Lung transcriptomic clock predicts premature aging in cigarette smoke-exposed mice

**DOI:** 10.1186/s12864-020-6712-z

**Published:** 2020-04-09

**Authors:** Mohamed-Amin Choukrallah, Julia Hoeng, Manuel C. Peitsch, Florian Martin

**Affiliations:** Philip Morris International R&D, Quai Jeanrenaud 5, 2003 Neuchâtel, Switzerland

**Keywords:** Age prediction, Machine learning, Transcriptomics, Cigarette smoke exposure, Lung inflammation

## Abstract

**Background:**

Lung aging is characterized by a number of structural alterations including fibrosis, chronic inflammation and the alteration of inflammatory cell composition. Chronic exposure to cigarette smoke (CS) is known to induce similar alterations and may contribute to premature lung aging. Additionally, aging and CS exposure are associated with transcriptional alterations in the lung. The current work aims to explore the interaction between age- and CS- associated transcriptomic perturbations and develop a transcriptomic clock able to predict the biological age and the impact of external factors on lung aging.

**Results:**

Our investigations revealed a substantial overlap between transcriptomic response to CS exposure and age-related transcriptomic alterations in the murine lung. Of particular interest is the strong upregulation of immunoglobulin genes with increased age and in response to CS exposure, indicating an important implication of B-cells in lung inflammation associated with aging and smoking. Furthermore, we used a machine learning approach based on Lasso regression to build a transcriptomic age model that can accurately predict chronological age in untreated mice and the deviations associated with certain exposures. Interestingly, CS-exposed-mice were predicted to be prematurely aged in contrast to mice exposed to fresh air or to heated tobacco products (HTPs). The accelerated aging rate associated with CS was reversed upon smoking cessation or switching to HTPs. Additionally, our model was able to predict premature aging associated with thoracic irradiation from an independent public dataset.

**Conclusions:**

Aging and CS exposure share common transcriptional alteration patterns in the murine lung. The massive upregulation of B-cell restricted genes during these processes shed light on the contribution of cell composition and particularly immune cells to the measured transcriptomic signal. Through machine learning approach, we show that gene expression changes can be used to accurately monitor the biological age and the modulations associated with certain exposures. Our findings also suggest that the premature lung aging is reversible upon the reduction of harmful exposures.

## Background

Aging of multi-cellular organisms is a complex biological process involving various molecular alterations such as epigenetic [1], genetic [2], and transcriptional changes [3–6]. These changes are likely modulated by various environmental factors that can either induce premature aging [7] or decelerate the natural aging rate [8]. The individual rates of aging greatly differ within a population, leading to the concept that the biological age can be monitored by a plethora of metrics, including omics-based measurements [9–11], providing robust indicators of healthy aging.

Modulation of transcriptional patterns is a central step in cellular response to numerous cues, including those related to aging. Gene regulation is one of the most investigated biological processes in relation to aging and a number of diseases in both humans and model organisms. Over the last decade, a massive number of transcriptomes have been generated, primarily by microarrays [12], in order to identify markers of aging and to understand the underlying mechanisms of aging processes. These studies revealed a number of common features for age-related transcriptomic changes, including the upregulation [13, 14] of genes involved in inflammation and stress response and the downregulation of genes associated with extracellular matrix constitution and metabolism [15, 16].

In the lung, biological aging correlates with anatomical and structural changes, such as fibrosis [17, 18] and immune dysregulation [19], mainly characterized by the alteration of inflammatory cell composition and chronic low-grade inflammation that are associated with progressive functional alterations. In rats, age-related lung fibrosis correlates with an increase in peri-bronchial collagen deposition and a decrease in matrix metalloproteinases (MMP) activity concomitant with an increase of tissue inhibitors of MMP (TIMP-1 and TIMP-2, 15). A number of external factors can induce aging-like structural and transcriptional alterations. For example, irradiated young mice exhibit lung transcriptomic profiles similar to unirradiated older mice [20]. Similarly, infection of young mice by respiratory syncytial virus induces transcriptomic alterations similar to those observed in uninfected old mice [16].

Chronic exposure to cigarette smoke (CS) contributes to many age-associated lung diseases, such as chronic obstructive pulmonary disease (COPD) and lung cancer [21]. Furthermore, aging and the response to CS exposure share common mechanisms involved in lung pathogenesis, such as impairment of proteostasis and autophagy leading to cellular senescence [22], suggesting that CS may contribute to premature lung aging.

Age- and CS-related transcriptomic alterations in the lung have been previously investigated; however, a detailed analysis of the overlap between these 2 transcriptomic patterns from technically comparable datasets is missing. Here, we took advantage of highly standardized transcriptomics experiments where the impact of CS on the lung was investigated over periods of exposure ranging from 6 to 8 months [23–25]. Therefore, these datasets from the same experimental setup were analyzed to provide information about age- and CS-associated transcriptional changes.

The current work seeks to investigate the transcriptomic crosstalk between CS and aging in the murine lung, build a transcriptomic age predictor, and evaluate the effect of environmental factors on transcriptomic age. We hypothesized that deviations from the average aging rate may indicate perturbed aging processes.

We found a substantial overlap between age- and CS-regulated genes; among those, immunoglobulin genes were the most upregulated in response to both aging and CS exposure. Moreover, we derived an age prediction model from transcriptomes of sham (fresh air)-exposed mice. Strikingly, the majority of age predictor genes were deregulated by CS, further supporting a strong interaction between aging and CS exposure. In line with this observation, CS-exposed mice were predicted to be prematurely aged, whereas mice exposed to aerosols from heated tobacco products (HTPs) had age predictions similar to those of the sham-exposed mice. Smoking cessation or switching to HTPs reduced the predicted accelerated transcriptomic aging. Similarly, mice exposed to fibrogenic irradiation (public datasets [20]) were predicted to be prematurely aged in comparison with untreated mice, further supporting the robustness of our prediction model when applied to an independent set of data. Altogether, our results indicate that transcriptomic clocks are a valuable tool to monitor the impact of environmental exposures on biological aging.

## Results

### Datasets

To investigate the relationship between lung aging and CS exposure at the transcriptional level, we leveraged 3 lung transcriptomic datasets [23–25] generated from wild-type female C57BL/6 or apolipoprotein E-deficient (ApoE^−/−^) transgenic mice exposed to CS (3R4F reference cigarette), fresh air (sham), or HTP aerosols over 6 to 8 months. The protocols also included smoking cessation and switching from CS to HTPs. Hereafter, these studies will be named E-MTAB-5281 [23], E-MTAB-5280 [24], and E-MTAB-7444 [25], as summarized in Supplementary Figure 1. All of the mice used in these studies were 2 months old at the beginning of the exposure.

### Substantial overlap between CS- and age-related transcriptional alterations in the lung

**Fig. 1:**
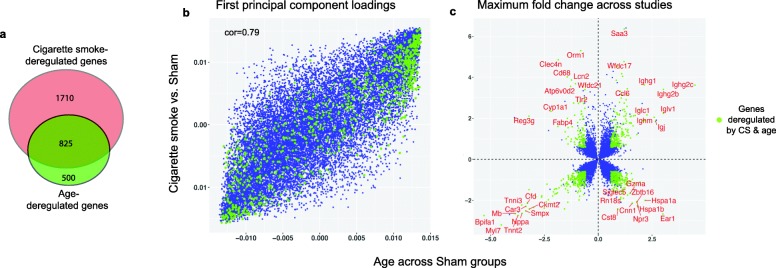
Substantial overlap between cigarette smoke (CS) exposure and aging at transcriptomic level. **a.** Venn diagram representing the overlap between age- and CS-regulated genes (false discovery rate [FDR] < 0.05 and log_2_ fold-change > log_2_(1.5). **b.** Comparison of the first principal component loadings for age- and CS-related gene expression changes across all datasets. Age-related changes were computed only for sham samples (e.g., sham_6m minus sham_1m, 28 such comparisons, inertia 30.3%). CS-related changes were computed by comparing 3R4F samples to the corresponding sham controls (e.g., 3R4F_6m minus sham_6m, 14 such comparisons, inertia 22.4%). **c.** Similar to B, the maximum fold change (MFC) across studies in relation to aging in sham groups (*x*-axis) is compared to MFC for 3R4F versus the corresponding sham control (*y*-axis). Genes commonly deregulated by aging and CS exposure on the basis of the FDR-adjusted *p*-value and fold change cut-offs (see Methods) are colored green in **b** and **c**. The top deregulated genes are labeled in red

Animals from the sham exposure groups were considered as representative of naturally aging mice and were used to calculate age-dependent differential gene expression. Age-regulated genes have been identified by comparing gene expression levels between sham samples from different age groups in each study (supplementary Table 1). In total, 1325 age-regulated genes were identified (see Methods). Strikingly, 62% of these genes were also deregulated by CS when compared to the corresponding sham controls from the same time points (Fig. 1a). This association was further supported by the strong correlation between the first principal component loadings derived from gene expression regulation profiles in response to aging and CS (Fig. 1b). Comparing the maximum values of gene expression fold changes (FC) associated with age and in response to CS across the 3 analyzed datasets showed strong directional interactions between CS- and age-related transcriptional responses (Fig. 1c). Of note, comparing maximum gene expression variations does not imply a statistical significance, nor does it indicate stable directional variations. Nevertheless, we observed a strong overlap between statistically significant differentially expressed genes (colored green in Fig. 1c) and genes with high maximum FC. Of particular interest, the top 10 genes co-upregulated by age and CS are central components of the immune response, and most of them are immunoglobulin genes (B-cell receptors), suggesting a substantial contribution of B-cell lineage in immune response to aging and CS exposure.

### Building a transcriptomic age predictor

It has been shown that DNA methylation levels of certain CpGs can be used to predict chronological age in humans [9, 11, 26, 27] and mice [28–32]; predictions in mice are generally less accurate than in humans and the most accurate DNA methylation clocks might not be optimal for evaluating aging modulations [29]. Transcriptomic age predictors have also been developed for human blood samples but are less accurate than their epigenetic counterparts [10]. To our knowledge, no transcriptomic age predictor has been developed for mice.

**Fig. 2:**
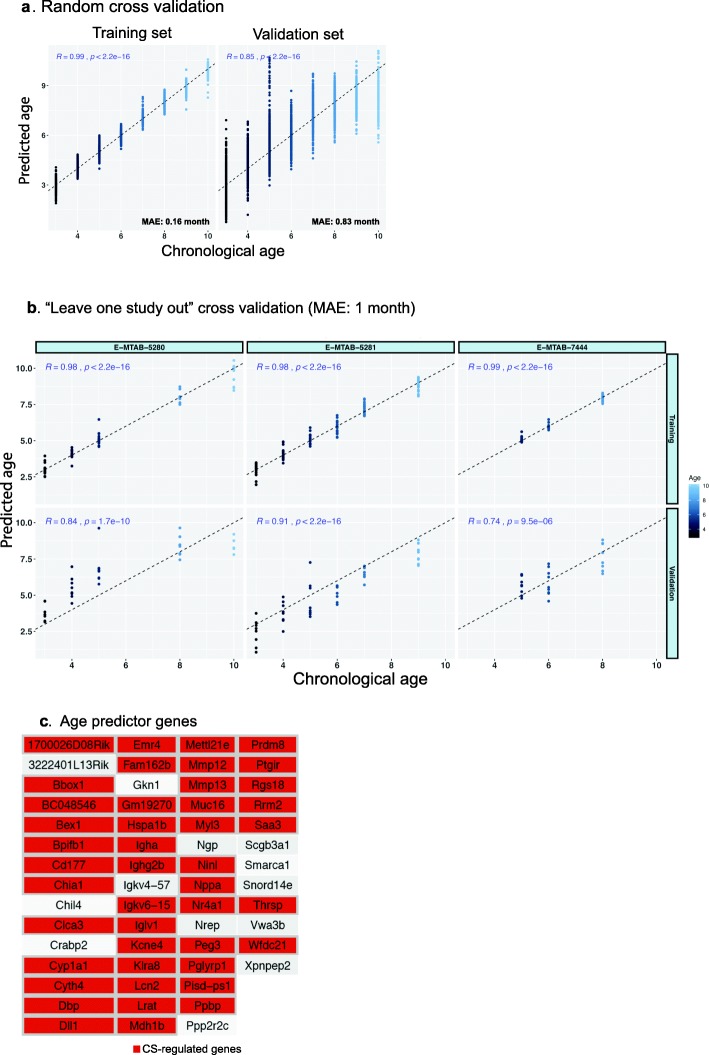
Development of age-prediction model from lung transcriptomics data (E-MTAB-5281, E-MTAB-5280 and E-MTAB-7444) using LASSO regression.. **a.** Random leave-out cross-validation over 100 runs. For every run, the 111 Sham samples from the 3 studies are randomly divided into training (75%) and validation sets (25%). **b**. Leave-one-study-out cross-validations. In each round, one study is excluded from the training but used for the validation. **c.** The final list of age predictor genes as selected by LASSO when applied to all of the 111 Sham transcriptomes. CS-regulated genes (see Methods) are colored red. MAE: mean average error

To test whether gene expression levels in murine lungs can be used to estimate chronological age, we used sham transcriptomes to derive an age regression model by applying the least absolute shrinkage and selection operator (LASSO) regression (see Methods). The performance of this prediction approach was evaluated by cross-validation, where sham samples were randomly partitioned into training and validation sets (Fig. 2a) and “leave one study out” cross-validation (Fig. 2b), resulting in a mean average error in the validation sets of 0.83 and 1 month, respectively, largely outcompeting the majority of mouse epigenetic clocks that are usually based on hundreds of regressors (CpGs). The final predictor derived by using all sham samples from the 3 analyzed datasets resulted in a set of 57 predictor genes. In line with our previous observations, the majority (44 out of 57) of those age predictor genes were also deregulated in response to CS exposure (Fig. 2c). Age predictor genes include key markers of CS exposure, such as Cyp1a1, Lcn2, and Mmp genes, in addition to genes involved in immune response, such as immunoglobulin genes.

### CS and thoracic irradiation accelerate lung transcriptional aging

**Fig. 3:**
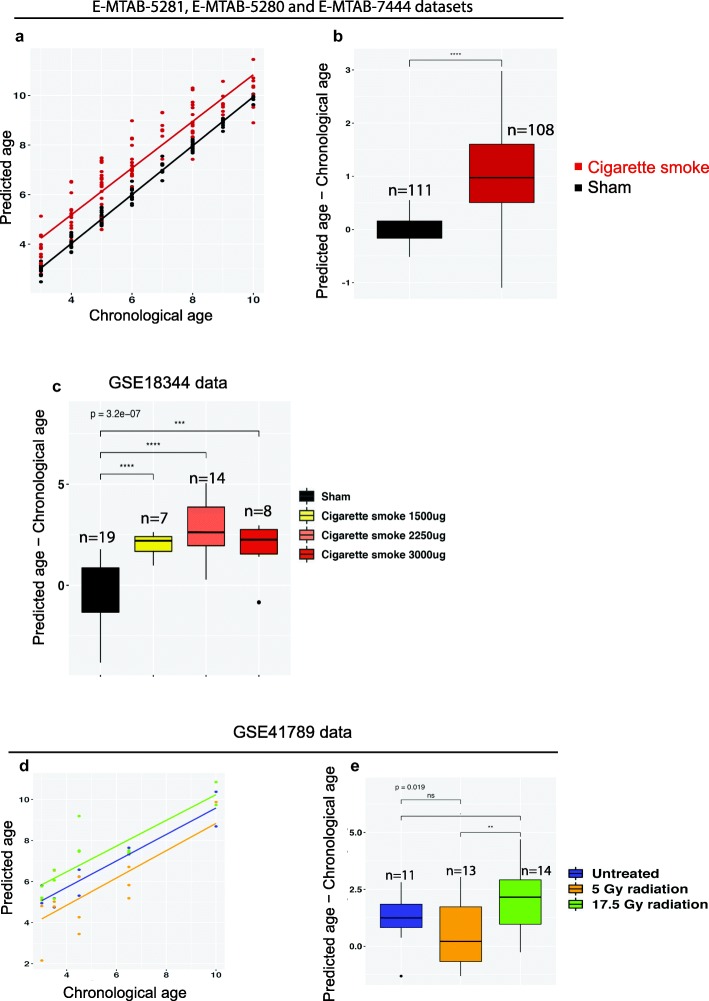
CS-exposed mice and highly irradiated mice are predicted to be prematurely aged. **a.** Chronological age versus predicted age for sham- and CS-exposed mice from E-MTAB-5281, E-MTAB-5280 and E-MTAB-7444 datasets. **b.** Boxplots summarizing the residuals (predicted age minus chronological age) for the same datasets. **c.** Age predictions of samples from an independent public dataset (GSE18344) where CD-1 mice where exposed to different daily doses of CS over 5 months. **d** and **e.** Prediction from the model applied to lung transcriptomes from the public dataset GSE41789. Age predictions were computed for 3 groups of mice; untreated mice and mice irradiated to 5 Gy or 17.5 Gy. Only 17.5 Gy induced lung fibrosis, senescence, and marks of accelerated aging according to the original study. Significance levels for t-test are indicated for each comparison (**** *P* < 0.0001; *** *P* < 0.001; ** *P* < 0.01; * *P* < 0.05;. *P* < 0.1; ns *P* > 0.1). The p-value from one-way anova test comparing all the groups is indicated

Given the strong transcriptional overlap between CS and aging, we hypothesized that CS exposure may affect the transcriptomic age predicted by the model above. To test this hypothesis, we applied the transcriptomic age predictor to CS-exposed samples. Although the model can accurately predict the age progression for those samples (Fig. 3a), predictions consistently showed a transcriptomic age higher than their chronological age (Fig. 3a and b). This result suggests that CS may contribute to premature lung aging, which is further supported by the overexpression of a number of immunoglobulin genes in CS-exposed samples when compared to their sham counterparts (Supplementary Figure. 2).

To test the performance of our model on independent datasets, we used publicly available transcriptomic lung data generated from 12 weeks CD-1 mice exposed to different daily doses of cigarette smoke (2R4F) over 5 months or to sham as a control [33]. The age predictor performed remarkably well in this dataset resulting in a clear distinction between fresh air and cigarette smoke-exposed samples (Fig. 3c). While the difference between chronological and predicted age was close to zero for sham samples, all CS-exposed samples were predicted to be prematurely aged (Fig. 3c).

To further evaluate the impact of other exogenous stimuli on the transcriptional age, we applied the age predictor to lung transcriptomes generated from mice exposed to different doses of thoracic irradiation (0 Gy, 5 Gy, and 17.5 Gy) [20]. The model accurately predicted age progression in the 3 experimental groups (Fig. 3c). Mice exposed to a fibrogenic dose of irradiation (17.5 Gy) were predicted to be prematurely aged in our model compared to non-fibrogenic treatments (Fig. 3d and e), in agreement with the original observations made by Citrin et al., where the 17.5 Gy irradiation dose, but not the 5 Gy dose, induced lung fibrosis, cellular senescence, and reduced survival in addition to transcriptional variations mimicking natural aging in irradiated young mice [20]. Surprisingly, the 5 Gy-exposed mice were, on average, predicted to be younger than the untreated mice, but with an increased variability in the prediction outputs.

### Smoking cessation and switching to HTPs reduce CS-associated accelerated transcriptional aging

**Fig. 4:**
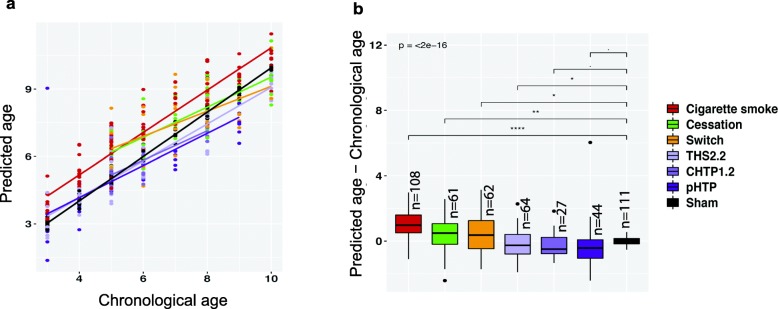
Smoking cessation and switching to HTPs reverse the accelerated transcriptomic aging. **a.** Chronological age versus predicted age for sham-, CS- and heated tobacco product-exposed mice as well as for cessation and switching groups. **b.** Boxplots of the residuals (predicted age minus chronological age) for the indicated groups. Significance levels for t-test are indicated for each comparison (**** *P* < 0.0001; *** *P* < 0.001; ** *P* < 0.01; * *P* < 0.05;. *P* < 0.1; ns *P* ≥ 0.1). The p-value from one-way ANOVA test comparing all the groups is indicated

We then evaluated the impact of smoking cessation or switching to potentially less harmful tobacco products on transcriptional premature aging. We applied our prediction model to transcriptomes generated from mice exposed to HTP aerosols under conditions matching exposure time and nicotine concentration of CS-exposed groups as well as to transcriptomes from cessation and switching groups (Supplementary Figure 1). At early time points, the transcriptomic age for cessation and switching groups was close to that of mice continuously exposed to CS and rapidly decreased to reach the range of age predictions for sham-exposed mice (Fig. 4a). This suggests that the CS-associated premature aging can be attenuated upon smoking cessation or switching to reduced-exposure products. In contrast to CS, chronic exposure to HTP aerosols correlates with weak deviations of the predicted age from the chronological age (Fig. 4a and b).

## Discussion

Modulation of gene expression is an important aspect of cellular adaptation to external factors, such as environmental exposures, but also to intrinsic physiological cues, including those associated with natural aging. The gene expression pattern measured at a given time reflects the cellular response to various concurrent stimuli, and this response is very likely to be affected by past events that might have produced stable genetic or epigenetic alterations. Thus, decoupling transcriptional changes associated with age from those associated with environmental exposures is challenging. Long-term exposure studies using animal models provide an ideal experimental setup to independently investigate transcriptional variations in response to different exposures as well as the variations associated with natural aging. Such investigations require large datasets generated with standardized procedures and covering a reasonable age range.

In the current work, we aimed to identify key transcriptomic markers for lung aging and investigate a potential association with CS exposure using three publicly available transcriptomic datasets generated from murine lung tissue exposed to CS and HTPs. Our investigations revealed a strong overlap between age- and CS-regulated genes in the lung. Age- and CS-associated transcriptional changes follow the same direction for some genes and adopt an opposite direction for others, demonstrating the complexity of transcriptional interactions between aging and exposure effects. One of the most striking observations was the strong upregulation of immunoglobulin genes, expression of which is exclusively restricted to B-cell lineage. This observation suggests that when exposed to CS or with increased age, the lung parenchyma is infiltrated by a significant number of B-cells. This observation is in line with previous studies reporting the presence of various B-cell structures in inflamed lungs. B-cell-rich lymphoid follicles were demonstrated in lung parenchyma of mice exposed to CS and in COPD patients with emphysema [34]. B-cells have also been reported to be present as part of the germinal centers within the parenchyma of inflamed mouse lungs and to produce local immunoglobulin E (IgE) isotype in response to antigenic challenge [35]. CS exposure has been shown to increase the volume of inducible bronchus-associated lymphoid tissue (iBALT) in aged mice in contrast to young mice [36] further supporting a higher susceptibility of aged tissues to environmentally induced inflammation. The iBALT volume seems to increase with age independently of CS exposure [36] suggesting that CS enhances a naturally occurring iBALT formation. Consistent with these results, the B-cell chemoattractant Cxcl13, known to be involved in lymphoid neogenesis in COPD [37] is upregulated with increased age and in response to CS exposure in our model (Supplementary Figure 2).

Further investigations of lymphoid infiltrates in aged lungs are required to better understand the contribution of B-cells to the age-associated inflammation. At the analytical level, the high expression of B-cell-specific genes in lung transcriptomes raises the question about the contribution of infiltrating immune cells to the measured gene expression or other molecular signals in the investigated tissues.

Over the last decade, a number of age predictors have been derived from methylomes [9, 27] transcriptomes [10, 38] and proteomes [39, 40]. These omics-based models sought to select a set of elements (e.g., CpGs, genes, proteins, etc.) that may predict chronological age and, eventually, the deviations associated with external factors. Although these reports provide valuable information about age-related molecular changes, they suffer from a number of limitations, including the interference of environmental factors and technical variabilities due to different platforms.

In the current work, we built an age predictor using a technically homogenous set of transcriptomes generated from untreated mice to minimize exposure and technical variability biases. We applied the LASSO regression algorithm that selects a limited number of regressor variables (genes) required for an optimal association with the response variable (age). This approach resulted in a set of 57 genes that can predicted chronological age with an average error of 0.83 months in the validation set. Interestingly, the majority of the age predictor genes were deregulated by CS, further supporting a strong connection between CS exposure and lung aging.

The predicted transcriptomic age from healthy untreated mice showed a high correlation with chronological age and can therefore be considered as a surrogate of natural biological age. Deviations from this standard aging curve may reflect perturbations in the natural aging rate. A positive delta age (predicted age minus chronological age) can be indicative of premature aging, whereas a negative delta age may indicate decelerated aging. To test this hypothesis, we applied our age predictor to transcriptomes from CS, HTPs, cessation, and switching groups. CS-exposed mice showed a consistent positive delta age, suggesting premature lung aging, in contrast to HTP-exposed mice, that showed a weak deviation from chronological age. Interestingly, transcriptomic age for cessation and switching groups rapidly deviated from that of CS-exposed group to reach that of the sham group.

Our prediction model was able to accurately predict age progression from an independent set of lung transcriptomes generated from mice irradiated to 5 Gy or 17.5 Gy and their untreated counterparts. Although we observed a positive delta age for the 3 experimental groups, the fibrogenic irradiation dose (17.5 Gy) had the highest predicted age, in line with the original conclusions made by Citrin et al., where the 17.5 Gy irradiation induced fibrosis and age-related transcriptomic alterations. By contrast, the untreated and 5 Gy-irradiated mice showed a weak delta age.

## Conclusions

The current work presents a comprehensive characterization of transcriptional interactions between cigarette smoke exposure and lung aging. We have provided a proof of concept that a machine learning approach applied to a technically homogenous set of transcriptomes can be used to model natural aging progression and detect deviations associated with environmental exposures. We also demonstrate the contribution of infiltrating immune cells to the measured gene expression in the investigated tissues. Additionally, we show that reducing harmful exposures may attenuate premature aging of lung tissue.

## Methods

### Microarray data processing

Raw data files were processed with the custom Chip Description File environment Mouse4302_Mm_ENTREZG v16.0 [41] and normalized using frozen robust microarray analysis [42].

### Identification of differentially expressed genes

Differentially expressed genes were identified based on the Benjamini–Hochberg FDR-adjusted *p*-values (cutoff 0.05) and fold changes (cutoff log_2_(1.5)) generated for the control-case contrasts with the *limma* R-package [43]. To define gene expression changes over age, expression values at a given time point were compared to those from all other time points within the same study for sham-exposed samples. For each contrast, the fold change is calculated as the expression value (log_2_ scale) in the oldest group minus the expression value in the youngest group (i.e., E-MTAB-5280 Sham_8m minus E-MTAB-5280 Sham_1m). Age-regulated genes were identified as genes meeting fold change and *p*-value criteria in at least one contrast. Given that 2 datasets used in this work are generated from ApoE^−/−^ mice, the ApoE gene was excluded from the entire analysis.

### Construction of transcriptomic age predictor

A LASSO model (implemented in the R-package *glmnet* [44]) was used to model chronological age (response variable) as a function of gene expression values (predictor variables) from sham samples. Lasso is a regression method that aims at selecting a limited subset of predictor variables through regularization while minimizing the prediction errors [45]. During the training step, the LASSO algorithm selects a set of regressors (genes) that optimally predict the dependent variable (age) via several rounds of internal cross-validations. LASSO is known to exclude correlated and non-informative regressors, thereby reducing the size of the predictor set. The set of genes used as age predictors was selected based the regularization parameter, leading to the minimum mean squared error value upon internal 5-fold cross-validations. The final model consisted of a linear regression of age as a function of the expression values of predictor genes selected by LASSO.

## Supplementary information

**Additional file 1: Figure S1.** Study design for the 3 datasets used in the current work. Sham corresponds to fresh air and is used as the exposure control. 3R4F corresponds to the standard reference cigarette, THS 2.2 and CHTP 1.2 correspond to candidate HTPs, and pHTP corresponds to prototype HTP. THS 2.2, Tobacco Heating System 2.2; CHTP 1.2, Carbon Heated Tobacco Product 1.2. The tables list the number of samples per experimental group; “m” stands for months of exposure. CS, cigarette smoke. ArrayExpress identifiers are indicated. All these studies used female mice.

**Additional file 2: Figure S2.** Mean gene expression values for Ighg2b, Ighg1, Igha, and Cxcl13 genes to illustrate the expression profile of B-cell-associated genes during aging. “m” in *x*-axis labels stands for months and indicates the exposure time. All animals were 2 months old at the beginning of the exposure. Error bars represent the standard error of the mean. Significance levels for ANOVA test are indicated for each group (**** *P* < 0.0001; *** *P* < 0.001; ** *P* < 0.01; * *P* < 0.05; . *P* < 0.1; ns *P* ≥ 0.1)

**Additional file 3.**

## Data Availability

The datasets analyzed during the current study are available in the ArrayExpress and GEO repositories: https://www.ebi.ac.uk/arrayexpress/experiments/E-MTAB-5281 https://www.ebi.ac.uk/arrayexpress/experiments/E-MTAB-5280 https://www.ebi.ac.uk/arrayexpress/experiments/E-MTAB-7444 https://www.ncbi.nlm.nih.gov/geo/query/acc.cgi?acc=GSE41789 https://www.ncbi.nlm.nih.gov/geo/query/acc.cgi?acc=GSE18344

## Abbreviations

ApoE−/−: Apolipoprotein E-deficient
CHTP 1.2: Carbon Heated Tobacco Product 1.2
COPD: Chronic obstructive pulmonary disease
CS: Cigarette smoke
FC: Fold changes
FDR: False discovery rate
HTP: Heated tobacco product
iBALT: Inducible bronchus-associated lymphoid tissue
Ig: Immunoglobulin
LASSO: Least absolute shrinkage and selection operator
MFC: Maximum fold change
MMP: Matrix metalloproteinases
THS 2.2: Tobacco Heating System 2.2
