# Supplementary material for: Lung transcriptomic clock predicts premature aging in cigarette smoke-exposed mice
Source: BMC Genomics. 2020 Apr 9;21:291. doi: 10.1186/s12864-020-6712-z (PMC7147004; doi:10.1186/s12864-020-6712-z)
Supplement: Supplementary file 1 — Additional file 1: Figure S1. Study design for the 3 datasets used in the current work. Sham corresponds to fresh air and is used as the exposure control. 3R4F corresponds to the standard reference cigarette, THS 2.2 and CHTP 1.2 correspond to candidate HTPs, and pHTP corresponds to prototype HTP. THS 2.2, Tobacco Heating System 2.2; CHTP 1.2, Carbon Heated Tobacco Product 1.2. The tables list the number of samples per experimental group; “m” stands for months of exposure. CS, cigarette smoke. ArrayExpress identifiers are indicated. All these studies used female mice. [file 12864_2020_6712_MOESM1_ESM.docx]

**Supplementary Figure 1.** Study design for the 3 datasets used in the current work. Sham corresponds to fresh air and is used as the exposure control. 3R4F corresponds to the standard reference cigarette, THS 2.2 and CHTP 1.2 correspond to candidate HTPs, and pHTP corresponds to prototype HTP. THS 2.2, Tobacco Heating System 2.2; CHTP 1.2, Carbon Heated Tobacco Product 1.2. The tables list the number of samples per experimental group; “m” stands for months of exposure. CS, cigarette smoke. ArrayExpress identifiers are indicated. All these studies used female mice.
