# Supplementary material for: Lung transcriptomic clock predicts premature aging in cigarette smoke-exposed mice
Source: BMC Genomics. 2020 Apr 9;21:291. doi: 10.1186/s12864-020-6712-z (PMC7147004; doi:10.1186/s12864-020-6712-z)
Supplement: Supplementary file 2 — Additional file 2: Figure S2. Mean gene expression values for Ighg2b, Ighg1, Igha, and Cxcl13 genes to illustrate the expression profile of B-cell-associated genes during aging. “m” in x-axis labels stands for months and indicates the exposure time. All animals were 2 months old at the beginning of the exposure. Error bars represent the standard error of the mean. Significance levels for ANOVA test are indicated for each group (**** P < 0.0001; *** P < 0.001; ** P < 0.01; * P < 0.05; . P < 0.1; ns P ≥ 0.1) [file 12864_2020_6712_MOESM2_ESM.docx]

**Supplementary Figure 2.** Mean gene expression values for Ighg2b, Ighg1, Igha, and Cxcl13 genes to illustrate the expression profile of B-cell-associated genes during aging. “m” in *x*-axis labels stands for months and indicates the exposure time. All animals were 2 months old at the beginning of the exposure. Error bars represent the standard error of the mean. Significance levels for ANOVA test are indicated for each group (**** P < 0.0001; *** P<0.001; ** P<0.01; * P<0.05; . P<0.1; ns P$\geq$0.1)
